# Income Tax by Physicians

**Published:** 1864-11

**Authors:** 


### INCOME TAX BY PHYSICIANS.

It will be remembered that we published some statistics, not long since based upon the returns of 1862. These returns were then private with the revenue office, and nothing but the most general facts were furnished; since that time the record has been opened to the public. It now appears that there has been great gain, and that the profession in this district are much better paid for their services. There is not, however, so great differences as might at first appear. Physicians are now charging higher for services, and the physician receiving the largest income was sick and absent, in 1862, so much as to materially lessen the amount he received. For 1863 fourteen physicians and one irregular practitioner pay upon over $1,000; of this number the lowest amount returned is $2,222; the highest amount is $10,438. It is gratifying to be able to show that physicians are appreciated and paid, while irregular practitioners, though nearly double the number of physicians, are still doing comparatively nothing, only one of the whole number paying upon $1,000 or over of professional income. It would appear from the statements of the irregulars themselves, and their friends, that their business was immense, but if they are to be believed when under oath, it is almost nothing—they actually receive for their services about what they are worth. In most large cities the charlatan and impostor are supposed to receive the highest premiums upon their tricks. Buffalo pays its own impostors poorly; but we mistrust that it contributes largely to imported and portable tricksters.

